# A periodic pumping technique of soil gas for ^222^Rn stabilization in large calibration chambers: part 2—theoretical formulation and experimental validation

**DOI:** 10.1038/s41598-020-71872-4

**Published:** 2020-10-06

**Authors:** Trilochana Shetty, Y. S. Mayya, K. Sudeep Kumara, B. K. Sahoo, B. K. Sapra, N. Karunakara

**Affiliations:** 1 Centre for Advanced Research in Environmental Radioactivity (CARER), Mangalore University, Mangalagangothri, Mangalore, 574 199 India; 2 Department of Chemical Engineering, IIT-Bombay, Mumbai, 400 076 India; 3 Radiological Physics and Advisory Division, Bhabha Atomic Research Centre (BARC), Trombay, Mumbai, 400 085 India

**Keywords:** Environmental sciences, Physics

## Abstract

In an adjoining publication, we demonstrated the novel technique to harvest soil gas of natural origin as a highly efficient source of ^222^Rn for calibration applications in a large volume ^222^Rn calibration chamber. Its advantages over the use of conventional high strength ^226^Ra sources, such as the capability to serve as a non-depleting reservoir of ^222^Rn and achieve the desired concentration inside the calibration chamber within a very short time, devoid of radiation safety issues in source handling and licensing requirements from the regulatory authority, were discussed in detail. It was also demonstrated that stability in the ^222^Rn concentration in large calibration chambers could be achieved within ± 20% deviation from the desired value through a semi-dynamic mode of injection in which ^222^Rn laden air was periodically pumped to compensate for its loss due to leak and decay. The necessity of developing a theory for determining the appropriate periodicity of pumping was realized to get good temporal stability with a universally acceptable deviation of ≤ ± 10% in the ^222^Rn concentration. In this paper, we present a mathematical formulation to determine the injection periods (injection pump ON and OFF durations) for the semi-dynamic operation to achieve long term temporal stability in the ^222^Rn concentration in the chamber. These computed pumping parameters were then used to efficiently direct the injection of soil gas into the chamber. We present the mathematical formulation, and its experimental validations in a large volume calibration chamber (22 m^3^). With this, the temporal stability of ^222^Rn concentration in the chamber was achieved with a deviation of ~ 3% from the desired value.

## Introduction

Inhalation of ^222^Rn, ^220^Rn and its decay products accounts for more than half of the annual effective dose from radiation sources of natural origin. Latest study carried out in Europe showed that residential ^222^Rn accounts for about 9% of the deaths from lung cancer and 2% of all cancer deaths^1^. Measurements and dose assessments due to ^222^Rn and progeny are performed using a wide variety of active and passive detectors and dosimeters. To set up standard protocols and maintain mutual conformity between the various detectors and instruments used by different laboratories, calibration facilities are established in different countries^1–3^.

Testing and calibration of ^222^Rn detectors in large volume walk-in type calibration chambers are carried out, mainly by three operational modes: (1) dynamic mode—in which ^222^Rn rich air from the source is continuously pumped to the chamber through the inlet and simultaneously maintaining the outlet in the open condition to attain the concentration levels^3–5^, (2) static mode—in which ^222^Rn laden air is filled once to achieve desired initial concentration value and the experiment is performed during the decay of ^222^Rn^5–7^, and (3) semi-dynamic mode—in which, initially ^222^Rn is injected into the chamber to obtain the desired value and then switched over to a pulsed mode of injection so that concentration is maintained within a certain band of the desired value. The periodicity of ^222^Rn injection is adjusted in such a way that the loss of ^222^Rn due to leak and decay is just compensated^8,9^. Calibration exercise at a low level (~ 1,000–5,000 Bq m^−3^)^10–12^ demand a steady ^222^Rn concentration for considerably long-time durations, from a few weeks to a few months.

In our previous publications, we demonstrated the harvesting of soil gas as a highly efficient secondary source of ^222^Rn for calibration experiments^13–16^ as well as a substitute for ^226^Ra source in research studies^17,18^. Advantages of soil gas ^222^Rn over the conventional sources are instantaneous natural availability, zero regeneration time required, zero cost, and excellent source stability. These features of soil gas ^222^Rn are well described in the previous publications^10,14,18^. Through the semi-dynamic mode of operation, it was shown that the ^222^Rn concentration could be maintained with a deviation of ± 20% from the desired value using soil gas. This deviation from the desired value is large since universally recommended long term calibration exposures demand a steady ^222^Rn concentration with an acceptable deviation of ≤  ± 10% for considerably long-time durations, from a few weeks to a few months for a large calibration chamber^11,12,19^. Higher deviation than the acceptable limit was because the periodicity of pumping was selected (1) by monitoring the concentration in the chamber using active monitors which may have some delayed response, (2) based on the knowledge of soil gas ^222^Rn concentration and the chamber volume, and (3) total ^222^Rn outflow from the chamber (sum of leak and decay)^8^ and not based on a theoretical model involving the functional behaviour of various parameters affecting the ^222^Rn concentration profile in the calibration chamber.

In this paper, we present a mathematical formulation to determine the exact injection periods (Pump ON and OFF durations) for ^222^Rn laden air for the semi-dynamic operation to achieve good long-term temporal stability in the ^222^Rn concentration in the chamber. These computed pumping parameters are then used to efficiently direct the injection of soil gas into the chamber. The experimental validations of the theoretical model and achievement of very good stability in ^222^Rn concentration in the chamber are discussed here.

## Mathematical formulation of the model and solutions

In the previous study^10^, it was demonstrated that the average value of natural ^222^Rn concentration in soil gas, measured over one full year, in the premises of the Centre for Advanced Research in Environmental Radioactivity (CARER) was 78.0 ± 20.0 kBq m^−3^. The average value of ^226^Ra activity concentration in soil was 42.0 ± 4.2 Bq kg^−1^.

Since soil gas ^222^Rn concentration is significantly larger than the concentration to be maintained, for a semi-dynamic mode of operation it is required to determine the optimum pumping rate that yields highest ^222^Rn stability over time at a predetermined concentration level. To achieve this, we set up ^222^Rn concentration evolution equation considering periodic injection combined with various removal processes. Let us consider the process in which ^222^Rn is injected for a time *T*_1_ and the injection was turned OFF for a period *T*_2_. Let *T* (= *T*_1_ + *T*_2_) be the duration of ON/OFF cycle. Since the injection is carried out through a flow, it is assumed that the flow in the chamber exists for the period *T*_1_ (it is zero during *T*_2_). During the period *T*_2,_ only leak out of the chamber and radioactive decay processes are removal mechanisms. As a result, we can analyse the process in terms of the injection cycles denoted by "*n*" and time *t* (*t,* 0 < *t* < *T*) within each cycle. The running time corresponding to a time *t* of the *n*th cycle may be represented as follows:

$${\text{Running time}} = nT + t,\quad {\text{where}}\;0 < t \le T\;{\text{and}}\; n = 0,1, 2, \ldots$$

Since pumping exists for time *T*_1_ and is zero for the time $T_{2} = T - T_{1}$ within each period, the corresponding time-dependent flow rate *f*(*t*) has the following form:

1a $$f(t) = f_{0} ,\quad for\; 0 < t \le T_{1}$$

1b $$f(t) = 0,\quad for\;T_{1} < t \le T$$

Let *A*_*n*_(*t*) denote the total ^222^Rn activity contained in the chamber in the *n*th cycle at time *t*. From simple mass balance consideration, the following equation may be set up for the evolution of *A*_*n*_(*t*):

2a $$\frac{{dA_{n} (t)}}{dt} = f_{0} C_{SG} - \left( {\frac{{f_{0} }}{V} + \lambda_{L} + \lambda_{R} } \right)A_{n} (t), \quad 0 < t \le T_{1} ;\;pumping\;on$$

2b $$\frac{{dA_{n} (t)}}{dt} = - \left( {\lambda_{L} + \lambda_{R} } \right)A_{n} (t),\quad T_{1} < t \le T;\; pumping\;off$$

where $V$: chamber volume (22.7 m^3^); $f_{0}$: pumping flow rate (m^3^ s^−1^); $f_{L}$: leak equivalent flow rate (m^3^ s^−1^); $C_{SG}$: soil gas ^222^Rn concentration at the inlet of the chamber (Bq m^−3^); $\lambda_{R}$: ^222^Rn decay constant (2.1 × 10^−6^ s^−1^); $\lambda_{L}$: chamber leak rate constant (s^−1^).

**Figure 1:**
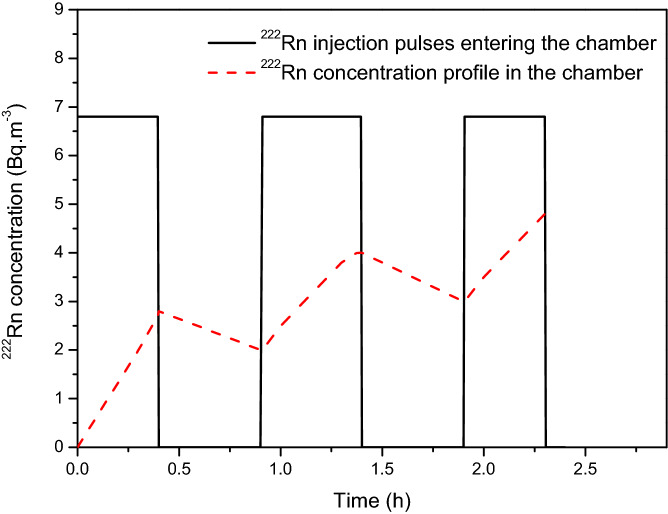
Demonstration sequence of ^222^Rn stabilization through periodic injection. Rectangles represent the concentration levels of the periodic injection pulses of ^222^Rn entering the chamber and dashed line represents the corresponding variation of the concentration profile in the chamber.

A schematic of the rise and fall of total ^222^Rn activity in the chamber as a function of pumping cycles is shown in Fig. 1. The hatched rectangular pulses are ^222^Rn injection rate sequences during the pumping process. The lines indicate an increasing and decreasing sequence of total air activity of ^222^Rn in the chamber due to injection and decay respectively. For general flow rates and periods of injection, the activity in the chamber will either go on increasing or decreasing over long periods. However, there exists an optimum ON–OFF ratio at which it will maintain a constant average value modulated by small ripples in every cycle.

**Figure 2:**
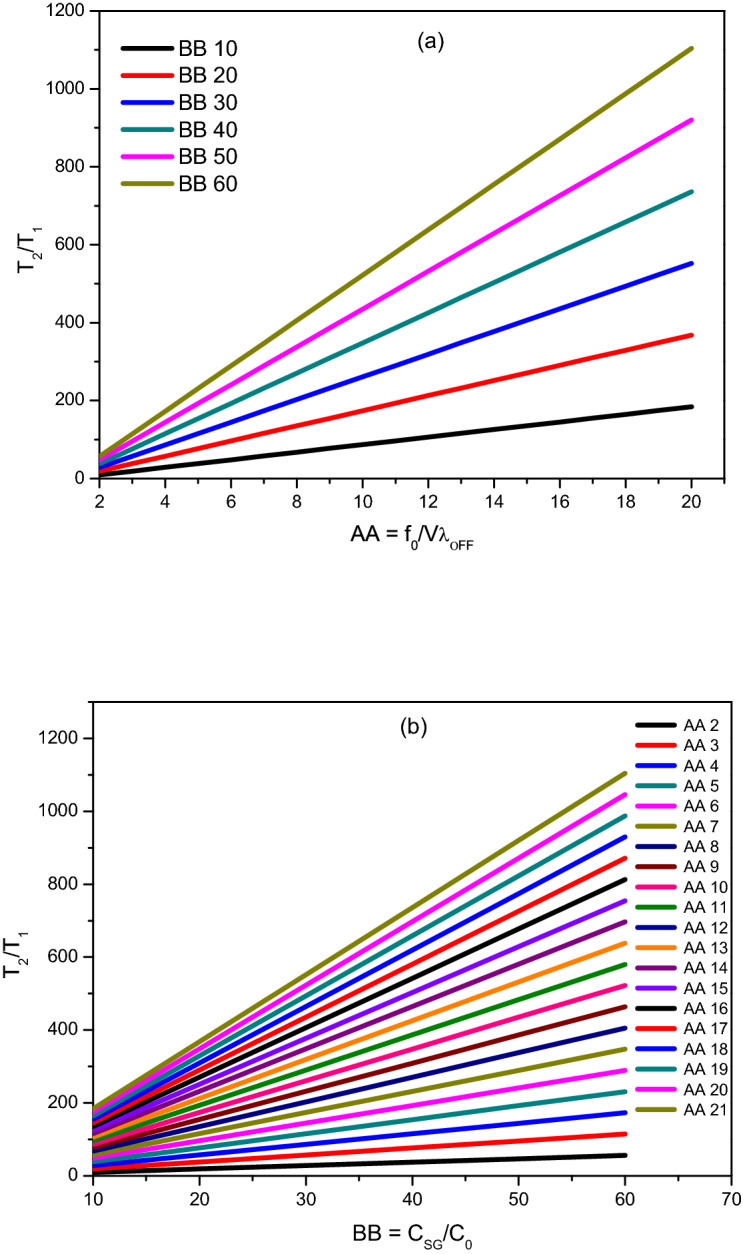
Theoretical plots of the variations of $\frac{{T_{2} }}{{T_{1} }}$, (**a**) with concentration ratio $BB = \frac{{C_{SG} }}{{C_{0} }}$ for different values of *AA*, (**b**) with leak rate ratios $AA = \frac{{f_{0} }}{{V\lambda_{OFF} }}$ at a different value of *BB*, as predicted by the model (Eq. 12b).

Equations (2a) and (2b) are a system of coupled first-order equations within each of the cycles. This system can be solved by specifying initial conditions at the beginning of the *n*th cycle. Let $A_{n} \left( 0 \right)$*,* as yet unknown, be the activity in the chamber at the beginning of *n*th cycle. We can now express the solution to Eqs. (2a, 2b) as follows:

3a $$A_{n} (t) = A_{n} (0)g_{1} (t) + \frac{{f_{0} }}{{\lambda_{ON} }}C_{SG} \left[ {1 - g_{1} (t)} \right],\quad 0 < t \le T_{1}$$

3b $$A_{n} (t) = A_{n} (0)g_{1} ( T_{ 1} )g_{2} (t) + \frac{{f_{0} }}{{\lambda_{ON} }}C_{SG} \left[ {1 - g_{1} (T_{1} )} \right]g_{2} (t),\quad T_{1} < t \le T$$

where we have introduced the concepts of “total removal rate” ($\lambda_{ON} )$ during pump flow ON condition and “total removal rate” ($\lambda_{OFF} )$ during pump OFF condition, defined as:

4a $$\lambda_{ON} = \left( {\frac{{f_{0} }}{V} + \lambda_{L} + \lambda_{R} } \right)$$

4b $$\lambda_{OFF} = \left( {\lambda_{L} + \lambda_{R} } \right)$$

Other definitions are:

5a $$g_{1} (t) = e^{{ - \lambda_{ON} t}} ,\quad 0 < t \le T_{ 1}$$

5b $$g_{2} (t) = e^{{ - \lambda_{OFF} \left( {t - T_{1} } \right)}} ,\quad T_{ 1} < t \le T$$

The unknown quantity $A_{n} (0)$ is determined by invoking the continuity conditions at the periodic boundaries. That is, the activity attained at the end of the *n*th cycle should be equal to the initial activity for the (*n* + 1)th cycle. i.e.

6a $$A_{n + 1} \left( 0 \right) = A_{n} \left( T \right)$$

Upon inserting Eq. (6a) in Eqs. (3a) and (3b), we obtain:

6b $$A_{n + 1} (0) = A_{n} (0)g_{1} (T_{ 1} )g_{2} (T) + \frac{{f_{0} }}{{\lambda_{ON} }}C_{SG} \left[ {1 - g_{1} (T_{ 1} )} \right]g_{2} (T),\quad n = 0, 1,2, \ldots$$

The initial condition for the iteration sequence is provided by the activity *A*_0_ injected for the first time (*n* = 0) into the chamber. i.e.

7 $$A_{0} (0) = A_{0}$$

Equation (6b) provides the iteration for obtaining $A_{n} (0)$ for all cycles. With this, one can write a recurrence relation in the following compact form:

8 $$A_{n + 1} (0) = \alpha A_{n} (0) + \beta \frac{{f_{0} }}{{\lambda_{ON} }}C_{SG} ,\quad n = 0,1, 2, \ldots$$

where

9a $$\alpha = g_{1} (T_{ 1} )g_{2} (T) = e^{{ - \lambda_{ON} T_{ 1} }} e^{{ - \lambda_{OFF} T_{ 2} }}$$

9b $$\beta = \left[ {1 - g_{1} (T_{ 1} )} \right]g_{2} (T) = \left( {1 - e^{{ - \lambda_{ON} T_{1} }} } \right)e^{{ - \lambda_{OFF} T_{2} }}$$

### Condition for stable concentration

To proceed further, we make an important assumption that the injected activity spreads rapidly in the chamber due to a mixing element such as fan, leading to spatially uniform ^222^Rn concentration. Detailed studies conducted with a 22.7 m^3^ calibration chamber at CARER^8,13^ with a mixing fan of capacity 3,620 m^3^ h^−1^ have shown that time required for attaining uniform concentration is < 10 min, which is much smaller than the pump OFF period (*T*_2_). With this experimental data we can convert the activities *A*_*n*_’s to concentrations *C*_*n*_’ as follows:

10a $$C_{n} = \frac{{A_{n} }}{V}$$

With this Eq. (8) can be rewritten as

10b $$C_{n + 1} \left( 0 \right) = \alpha C_{n} \left( 0 \right) + \beta \frac{{f_{0} }}{{V\lambda_{ON} }}C_{SG} ,\quad n = 0,1, 2, \ldots$$

In this periodic injection scenario, one cannot achieve perfectly uniform ^222^Rn concentrations because of the finite time required for homogenization within the chamber. However, the presence of a strong mixing element like a fan is expected to rapidly spread the injected gas throughout the chamber. As the chamber has a certain volume of outflow per hour (due to leak and decay) it is possible only to maintain a stable mean concentration. From Eq. (10b) we see that if the mean concentration has to remain constant right from the first cycle, it should not have a dependency on '*n’* Eq. (8) suggests that it is possible only when *C*_*n*_(0) remains independent of *n*. That means all *C*_*n*_(0)’s should be the same as the initial concentration *C*_0_ *i.e.*

11a $$C_{n} \left( 0 \right) = C_{0 } { }$$

Upon applying this condition to Eq. (8), we obtain.

i.e.,

$$C_{0} = \alpha C_{0} + \beta S_{0}$$

*i*.*e*.,

11b $$\frac{{S_{ 0} }}{{C_{ 0} }} - \frac{{\left( {1 - \alpha } \right)}}{\beta } = \frac{{g_{ 2} \left( T \right)\left[ {1 - g_{ 1} \left( {T_{ 1} } \right)} \right]}}{{1 - g_{ 1} \left( {T_{ 1} } \right)g_{ 2} \left( T \right)}}$$

It can be expressed elegantly as

11c $$e^{{\lambda_{OFF} T_{ 2} }} = e^{{ - \lambda_{ON} T_{ 1} }} + \frac{{f_{0} C_{SG} }}{{V\lambda_{ON} C_{ 0} }}\left[ {1 - e^{{ - \lambda_{ON} T_{ 1} }} } \right]$$

This is an exact equation which relates *T*_1_ (the injection period) to *T*_2_ (the pump OFF period) for a given set of the quantity $\frac{{f_{0} C_{SG} }}{{V\lambda_{ON} C_{ 0} }}$. This equation may be further simplified under the assumption that the period of pumping is much smaller than the mean residence times i.e. *λ*_*ON*_*T*_1_ << 1. This is justified because typically for a large chamber (in the present case 22.7 m^3^) *λ*_*ON*_ is controlled by the flow rate. For a flow rate of 60 L min^−1^, *λ*_*ON*_ is about 0.16 h^−1^ which is lesser than the frequency of pumping $\frac{1}{{T_{1} + T_{ 2} }}$. The injection period is determined by stipulating that the concentration deviation from the stipulated mean due to decay/leak during the OFF period should not exceed, say 5%. As explained in Sect. 3 (Fig. 5), typically, the frequency would be 1 min pumping after every 59 min. This will, of course, change depending upon the soil gas concentration and the concentration required in the chamber, as indicated in Eq. (12c). In such a case, the exponential terms can be approximated by linear terms and one obtains:

$$1 + \lambda_{OFF} T_{ 2} = 1 - \lambda_{ON} T_{ 1} + \frac{{f_{0} C_{SG} }}{{V\lambda_{ON} C_{ 0} }}\lambda_{ON} T_{ 1}$$

This results in the following formula:

12a $$\frac{{T_{2} }}{{T_{1} }} = \frac{{\lambda_{ON} }}{{\lambda_{OFF} }}\left( {\frac{{f_{0} C_{SG} }}{{V\lambda_{ON} C_{ 0} }} - 1} \right)$$

For future purposes, we denote this ratio by "*R*", where

12b $$R = \frac{{T_{2} }}{{T_{1} }} = \frac{{\lambda_{ON} }}{{\lambda_{OFF} }}\left( {\frac{{f_{0} C_{SG} }}{{V\lambda_{ON} C_{ 0} }} - 1} \right)$$

Since *λ*_*ON*_ is related to *λ*_*OFF*_ through flow rate, Eq. (12b) may be written as:

12c $$\frac{{T_{2} }}{{T_{1} }} = \frac{{f_{0} }}{{V\lambda_{OFF} }} \cdot \frac{{C_{SG} }}{{C_{0} }} - \left( {1 + \frac{{f_{0} }}{{V\lambda_{OFF} }}} \right)$$

Equation (12c) is the desired condition for attaining stable concentration right from the beginning with periodic injection. It is expressed as the ratio of duration of the pump OFF (*T*_2_) and pump “ON” (*T*_1_) periods in terms of two groups of system parameters, namely leak rate ratio

13a $$AA = \frac{{f_{0} }}{{V\lambda_{OFF} }},$$

and concentration ratio

13b $$BB = \frac{{C_{SG} }}{{C_{0} }},$$

**Figure 3:**
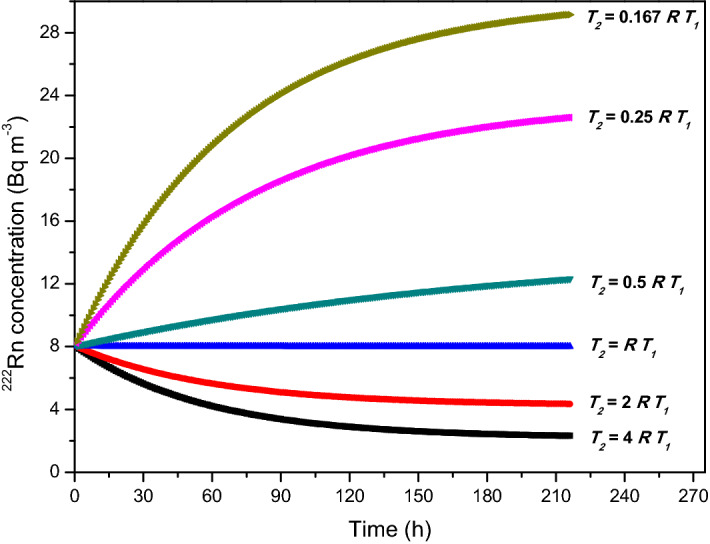
Illustration of how the concentration remains at the required level right from the start when the stabilization condition (Eq. 12b) is satisfied (blue line); Concentration increases (or decreases) to reach different steady-state levels when OFF period (*T*_2_) is less (or greater) than the optimal value, *RT*_1_*.*

It is important to note the following points:

- In most situations, *C*_*SG*_ >> *C*_0_ and $\frac{{f_{0} }}{{V\lambda_{OFF} }}$ >> 1, and hence $\frac{{T_{2} }}{{T_{1} }}$ >> 1. This means the fraction of OFF period is much larger than the fraction of ON period in a given injection cycle. In practice, Eq. (12c) is useful for fixing the OFF period (*T*_*OFF*_) for a given soil gas pumping ON period. Practical consideration (such as, the response time of the pump, the time required for the soil gas to reach from the point from where it is drawn to the pump through tubing which is maintained sufficiently long to minimise the ^220^Rn concentration) demands that the ON period cannot be too short say less than one minute. Hence one would prefer the ON period to be at least for about a minute. Once this is done, then the next injection should be done after time *T*_2_ = *R T*_1_ and the cycle period will naturally be *T* = (1 + *R*) *T*_1_ for concentration stabilization.
- The formula also offers us constraints on the highest concentration that can be stabilized for a given soil gas concentration and flow rate. For example, for continuous injection *T*_2_ = 0 and the concentration that gets stabilized is:
  $$C_{0} = \frac{{f_{0} }}{{f_{0} + V\lambda_{OFF} }}C_{SG}$$
  Figure 2(a,b) shows the variations of $\frac{{T_{2} }}{{T_{1} }}$ for concentration ratio *BB* and leak rate ratio *AA* at different flow rates. *T*_2_ range can vary over 100 times much than *T*_1_ if soil gas concentration is higher. There will be fluctuations or wiggles due to the periodicity of the injection process. We can decrease the wiggles by increasing the intervals of injection.
- It predicts that the ratio of the pump OFF (T_2_)/Pump ON (*T*_1_) time is related to the removal rates in the chamber with and without flows and the ratio of soil gas ^222^Rn concentration *C*_*SG*_ to the stipulated concentration *C*_0_ to be maintained in the chamber. However, it should be emphasized that Eq. (12c) can only predict the ratio of the times $\frac{{T_{2} }}{{T_{1} }}$ but not the total period (*T* = *T*_1_ + *T*_2_) of the cycle. To arrive at an optimal value for the period one has to stipulate additional constraints. For this, we impose that the fluctuations around the stabilized mean value lie within pre-specified limits and the variations of stabilized mean ^222^Rn concentration with values of R is shown below in Fig. 3. It illustrates how the stabilization is achieved when the system is operated at *T*_2_ = *RT*_1_; The concentration either overshoots or undershoots to higher or lower steady-state value when *T*_2_ < *RT*_1_ and *T*_2_ > *RT*_1_, respectively. The steady-state is reached as a result of an average balance between the injection rate and the decay + leak rates. It is observed from Fig. 3 that the stability of the concentration of ^222^Rn to be maintained in the chamber depends on the ratio of *T*_2_ and *T*_1_ which gives an optimized value of *R* = *R*_*optimum*_, which may be estimated from Eq. (12b). At this value of *R*, it achieves maximum stability followed by its deterioration for other orders of *R*_*optimum*_.

## Materials and method

### Set up for extraction and periodic pumping of soil gas

**Figure 4:**
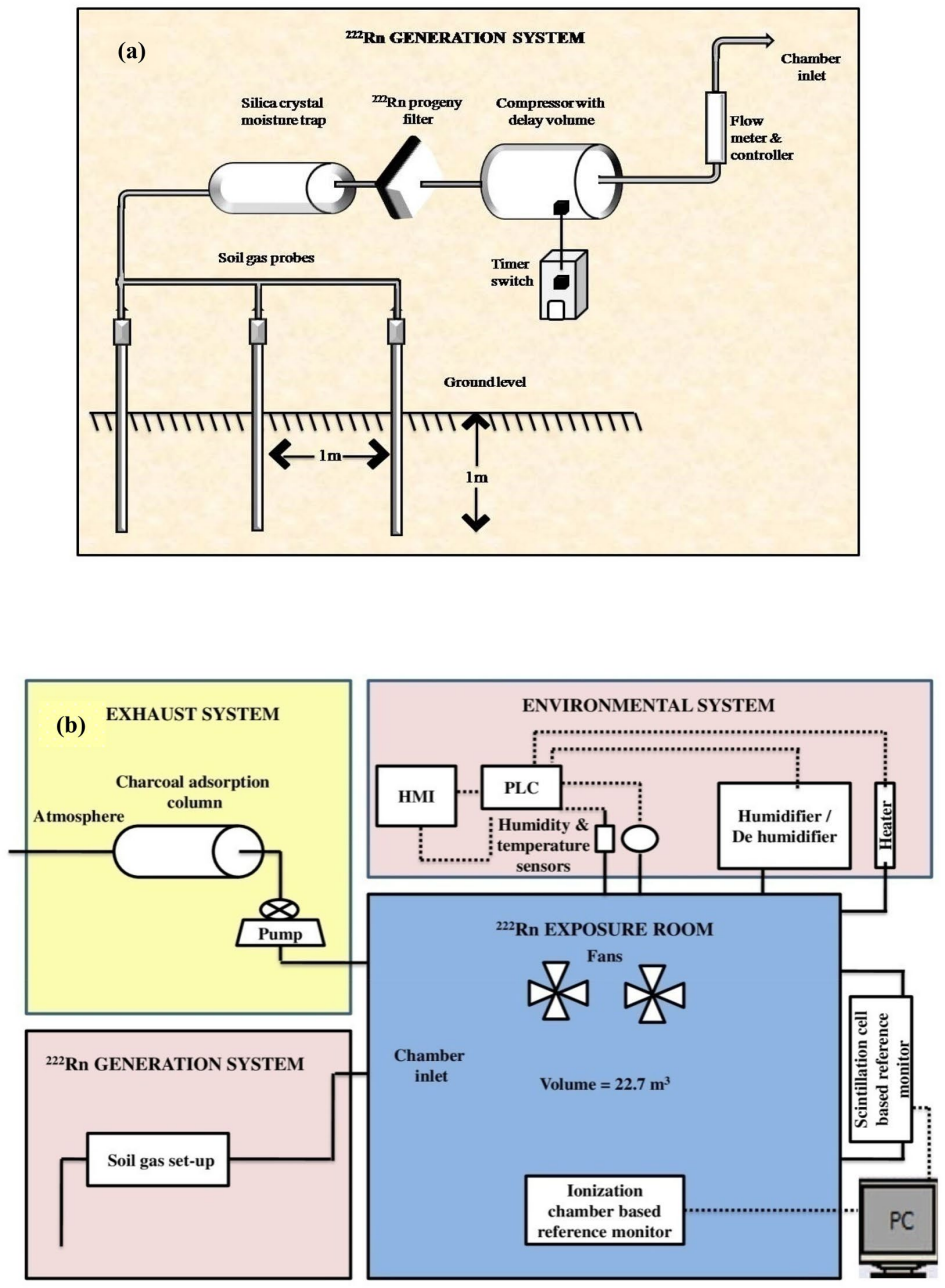
Schematic diagram of the (**a**) soil gas probe set up for drawing soil gas, and (**b**) block diagram of the ^222^Rn calibration chamber.

The details of soil gas extraction were described in a previous publication^10^. Since the ^222^Rn concentration in soil gas increases exponentially with depth and saturates to an equilibrium concentration at a depth greater than about 0.8 to 1 m^20,21^, it was extracted from a depth of about a meter. The extraction was performed using a hand-driven soil gas probe (STITZ, Germany) inserted inside the ground as shown in Fig. 4a. The total flow is bifurcated into multiple channels from each of which a probe is inserted into the soil and this is done to eliminate the possibilities of soil particles choking the inlet of the probes at higher flow rates. The separation between the probes was generally kept at a distance of about 1 m. The bifurcation reduces the suction velocity at the probe inlet and the possibility of large area perturbations of ^222^Rn concentration in the soil matrix. The outlets of multiple soil probes were combined and are connected in series to a progeny filter, a dehumidifier, and a buffer volume of 0.052 m^3^ (for mitigation of ^220^Rn present in the soil gas). The detailed schematic diagram of the calibration chamber and soil gas probe arrangement to draw soil gas is shown in Fig. 4b.

### Reference monitors

To monitor the ^222^Rn concentration inside the calibration chamber the reference instruments used for the present study were scintillation cell-based monitors (Smart RnDuo, AQTEK SYSTEMS, India) with detection range from 8 Bq m^−3^ to 50,000 kBq m^−3^. These monitors were periodically calibrated at Bhabha Atomic Research Centre (BARC), Mumbai once in every six months using a standard solid flow-through type ^226^Ra source of activity 110.6 kBq (uncertainty < 2%) (model RN − 1,025, Pylon electronics, Ottawa, Canada). The monitors were operated in flow mode with an operational cycle interval of 15 min during the experiments.

### Experimental setup for validation of the theory

The experimental validations of the proposed theory for maintaining temporal stability in ^222^Rn concentration in the calibration chamber was achieved with the help of a programmable timer switch, which controlled the switching ON/OFF of the pump used to draw soil gas from the probe insertion point. Initially, the soil gas was pumped into the chamber continuously till a desired ^222^Rn concentration was attained in the chamber. Once the desired ^222^Rn concentration level was attained, the pumping was switched over to “periodic pumping mode”. The duration of pumping (*T*_1_), the period of pumping cycle (*T*) and duration of pump OFF (*T*_2_= *T* − *T*_1_) were determined based on the ratio $\frac{{T_{2} }}{{T_{1} }}$ = *R* using Eq. (12c). The process of switching ON and OFF of the pump was performed automatically by the programmable timer switch to maintain temporal stability of concentration of ^222^Rn in the chamber. Volumetric average ^222^Rn concentration in the chamber was measured both during the transient build-up of initial desired level and periodic injections at an inlet port of the chamber, with a Smart RnDuo online ^222^Rn monitor. Besides, continuous monitoring of the concentration was also performed using two AlphaGuard systems placed inside the chamber at two different points at a height of 1 m from the floor of the chamber.

## Results and discussion

Stability of ^222^Rn concentration in the walk-in chamber was achieved by periodic injections of soil gas. The amount of ^222^Rn injected was equal to the sum of volume outflow of ^222^Rn per hour due to leak and decay. We demonstrate the experiments for the fan ON condition in particular for two reasons, (1) fan ON condition is the situation of maximum ^222^Rn depletion in the chamber due to the outward pressure exerted by air inside the chamber, (2) the circulation fan in the calibration chamber facility will always be switched ON to maintain spatial uniformity of ^222^Rn concentration, relative humidity (*RH*) and temperature during the calibration experiments.

**Figure 5:**
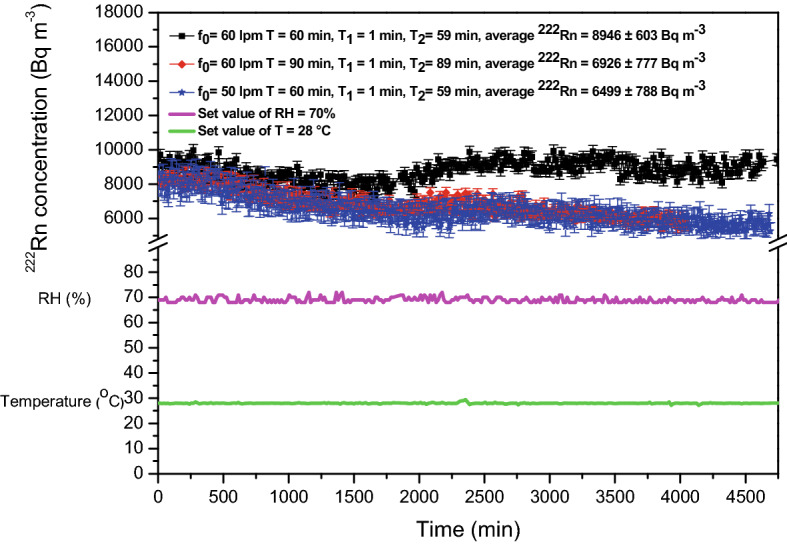
Temporal stability of ^222^Rn concentration attained in the chamber in fan ON condition for 5 days (at a set value of temperature = 28 °C, *RH* = 70%).

Initially, experiments were conducted by randomly selecting the duration and intervals of pumping. The experiments were performed for three different combinations of the period of pumping cycle *T*, pumping duration *T*_1_, non-pumping duration *T*_2_ and soil gas flow rates as given in Table 1. The ^222^Rn concentration in the chamber was raised to desired levels of 9,040 ± 294 Bq m^−3^, 8,330 ± 311 Bq m^−3^ and 8,096 ± 664 Bq m^−3^ (selected arbitrarily, the concentration achieved in the chamber after initial pumping of the soil gas was considered as the desired value for demonstration purpose) during the three different experiments. Once this desired concentration was attained, experiments were continued for a duration of ~ 5 days by periodic injections of soil gas as per the duration and intervals of pumping decided above. The temporal stability of ^222^Rn concentration attained with this method is shown in Fig. 5. Average values of ^222^Rn concentration of 8,946 ± 603 Bq m^−3^, 6,926 ± 777 Bq m^−3^ and 6,499 ± 788 Bq m^−3^ were achieved in the chamber during these experiments. Relative humidity and temperature inside the chamber were maintained at 70% and 28 °C during the experiment.

**Table 1:** Pumping parameters for periodic injections of soil gas into the chamber (parameters selected randomly).

Case No.	Soil gas pumping flow rate, f_0_ (L min^−1^)	Period of the pumping cycle, *T* (min)	Concentration needs to be maintained in the chamber, *C*_0_ (kBq m^−3^)	Duration of pumping, *T*_1_ (min)	Non-pumping duration, *T*_2_ (min)
Case 1	60	60	9.0	1	59
Case 2	60	90	8.3	1	89
Case 3	50	60	8.1	1	59

It was observed from the results obtained (Fig. 5) that the stability of ^222^Rn concentration in the chamber was better for case 1 (Table 1) in which the deviation observed from the desired concentration level was ~ 1% for a particular combination of *T, T*_1_*, T*_2_ and *f*_0_. But, for other combinations of pump ON and OFF (cases 2 and case 3) the average ^222^Rn concentration achieved in the chamber were 6,926 ± 777 Bq m^−3^ and 6,499 ± 788 Bq m^−3^ respectively which correspond to deviations of 17% and 20% from the desired values. Also, as evident from Fig. 5 the concentration values show a decreasing trend with time since the ^222^Rn pumped during the selected pumping period intervals was not able to compensate for decay and leak. This confirms the fact that one cannot choose a random combination of pumping parameters. Hence, for achieving good temporal stability of ^222^Rn concentration, an accurate pumping algorithm should be adopted.

In the theoretical model-based approach the parameters of pumping such as *T, T*_1_ and *T*_2_ for different soil gas flow rates were calculated using Eq. (12c). The experiments were carried out for different values of *R*, with an average soil gas ^222^Rn concentration, *C*_SG_, of 78.0 ± 20.0 kBq m^−3^ (2σ) and the flow rate, *f*_0,_ was fixed at 30 L min^−1^. For validation of the theory, let us stipulate that a particular calibration experiment is performed by maintaining a desired ^222^Rn concentration of ~ 8,000 Bq m^−3^. The details of the parameters of pumping, determined based on the theory developed in this study (Eq. 12c), for maintaining the desired concentration are presented in Table 2.

**Table 2:** Parameters for periodic injections of soil gas into the chamber (computed based on the theory developed, Eq. 12c).

Soil gas pump ON/OFF ratio, *R*		Total removal rate		Effective soil gas concentration, *S*_0_ (kBq m^−3^)	Concentration to be maintained in the chamber, *C*_0_ (kBq m^−3^)	Period of the pumping cycle, *T* (min)	Duration of pumping, *T*_1_ (min)	Non-pumping duration, *T*_2_ (min)
		Flow ON, *λ*_*ON*_ (s^−1^)	Flow OFF *λ*_*OFF*_ (s^−1^)					
*R/6*	12.70	2.44E−5	4.49E−6	67.63	8	60	4	56
*R/4*	19.01					60	3	57
*R/2*	38.06					30	1	29
*R*	76.00					60	1	59
*2R*	152.13					110	1	109

**Figure 6:**
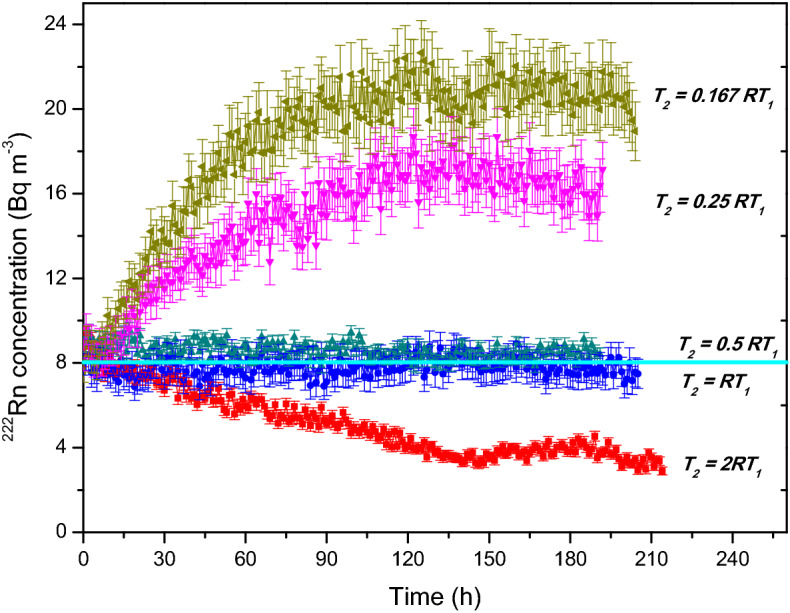
Experimentally achieved temporal stability in ^222^Rn concentration in the chamber for the fan ON condition for 9 days for variations in *R* (temperature = 28 °C, *RH* = 60%). The trend in the variation of concentration observed experimentally is exactly similar to the theoretical prediction (Fig. 3).

The temporal stability of ^222^Rn concentration achieved in the chamber, for the parameters given in Table 2, are presented in Fig. 6. As discussed earlier, the ratio *R* is an important parameter in deciding the temporal stability of concentration, and this is demonstrated in Fig. 6. Experimental results (Fig. 6) confirm the predictions of the theory (Fig. 3) for all values of *R*. As evident from the results, when *T*_2_ = *R T*_1_ (i.e. *R* = *R*_*optimum*_) excellent temporal stability in the concentration was achieved throughout the experimental duration of 9 days with an average value of  7782 ± 376 Bq m^−3^, which corresponds to a deviation of < 3% from the desired value. This deviation is well within the standard error in the measurements. For all the other values of R (*T*_2_ = *R*/6 *T*_1_, *R*/4 *T*_1_, *R*/2 *T*_1_ and 2*R T*_1_) there was either a build-up or a depletion of concentration in the chamber. These experimental results validate the predictions of the theory and the algorithm developed here will find immense application in large calibration chamber facilities for maintaining good temporal stability of ^222^Rn concentration.

To summarise, the theoretical model developed in the present study allows optimization of the pumping rate, pumping duration and pump ON/OFF time for achieving excellent long term stability in the desired concentration of ^222^Rn, with deviation ≤  ± 3%, in the chamber for calibration experiments. The technique is highly advantageous due to its technical simplicity and economic considerations. When coupled with the optimized periodic pumping algorithm the technique of harvesting of soil gas as an un-depleting source of ^222^Rn would eliminate the need for expensive radioactive sources.

## List of symbols

*AA*: Leak rate ratio
*BB*: Concentration ratio
$C_{0}$: Initial ^222^Rn concentration to be maintained in the chamber (Bq m^−3^)
$C_{SG}$: Soil gas ^222^Rn concentration (Bq m^−3^)
$C_{peak}$: Peak ^222^Rn concentration (Bq m^−3^)
$C_{n} \left( t \right)$: Concentration at time *t* of the *n*th cycle (Bq m^−3^)
$C_{n} \left( 0 \right)$: Concentration at the beginning of the *n*th cycle (Bq m^−3^)
$\overline{C}_{n}$: Mean ^222^Rn concentration in each cycle (Bq m^−3^)
$f_{0}$: Pumping flow rate (m^3^ s^−1^)
$f_{L}$: Leak equivalent flow rate (m^3^ s^−1^)
$f(t)$: Pumping flow rate at time *t* (m^3^ s^−1^)
$R$: Ratio of OFF/ON periods of soil gas pumping
$R_{Optimum}$: Ratio of OFF/ON periods of soil gas pumping for maximum concentration stability attainable
$S_{0}$: Effective soil gas concentration (Bq m^−3^)
$T$: Period of a pumping cycle (s)
$T_{2} = T - T_{1}$: Non-pumping duration (s)
$$V$$: Chamber volume (m^3^)
$\lambda_{R}$: ^222^Rn decay constant (s^−1^)
$\lambda_{L} = \frac{{f_{L} }}{V}$: Leak rate constant (s^−1^)
$\lambda_{ON} = \left( {\frac{{f_{0} }}{V} + \lambda_{L} + \lambda_{R} } \right)$: Total removal rate during pump ON period $T_{1}$ (s^−1^)
$\lambda_{OFF} = \left( {\lambda_{L} + \lambda_{R} } \right)$: Total removal rate during pump OFF period $T_{2}$ (s^−1^)
